# DiSC: a statistical tool for fast differential expression analysis of individual-level single-cell RNA-seq data

**DOI:** 10.1093/bioinformatics/btaf327

**Published:** 2025-05-30

**Authors:** Lujun Zhang, Lu Yang, Yingxue Ren, Shuwen Zhang, Weihua Guan, Jun Chen

**Affiliations:** Division of Biostatistics and Health Data Science, School of Public Health, University of Minnesota, Minneapolis, MN 55455, United States; Department of Quantitative Health Sciences, Mayo Clinic, Rochester, MN 55905, United States; Department of Quantitative Health Sciences, Mayo Clinic, Jacksonville, FL 32224, United States; Department of Quantitative Health Sciences, Mayo Clinic, Rochester, MN 55905, United States; Division of Biostatistics and Health Data Science, School of Public Health, University of Minnesota, Minneapolis, MN 55455, United States; Department of Quantitative Health Sciences, Mayo Clinic, Rochester, MN 55905, United States

## Abstract

**Motivation:**

Single-cell RNA sequencing (scRNA-seq) has become an important method for characterizing cellular heterogeneity, revealing more biological insights than the bulk RNA-seq. The surge in scRNA-seq data across multiple individuals calls for efficient and statistically powerful methods for differential expression (DE) analysis that addresses individual-level biological variability.

**Results:**

We introduced DiSC, a method for conducting individual-level DE analysis by extracting multiple distributional characteristics, jointly testing their association with a variable of interest, and using a flexible permutation testing framework to control the false discovery rate (FDR). Our simulation studies demonstrated that DiSC effectively controlled the FDR across various settings and exhibited high statistical power in detecting different types of gene expression changes. Moreover, DiSC is computationally efficient and scalable to the rapidly increasing sample sizes in scRNA-seq studies. When applying DiSC to identify DE genes potentially associated with COVID-19 severity and Alzheimer’s disease across various types of peripheral blood mononuclear cells and neural cells, we found that our method was approximately 100 times faster than other state-of-the-art methods and the results were consistent and supported by existing literature. While DiSC was developed for scRNA-seq data, its robust testing framework can also be applied to other types of single-cell data. We applied DiSC to cytometry by time-of-flight data, DiSC identified significantly more DE markers than traditional methods.

**Availability and implementation:**

The R software package “SingleCellStat” is freely available on CRAN (https://cran.r-project.org/web/packages/SingleCellStat/index.html) and GitHub (https://github.com/Lujun995/DiSC). The replication code for reproducing the analyses in this study is publicly accessible at https://github.com/Lujun995/DiSC_Replication_Code. The scRNA-seq expression matrix and metadata utilized in our simulations and analyses can be retrieved from https://cells.ucsc.edu/autism/rawMatrix.zip, https://cellxgene.cziscience.com/collections/1ca90a2d-2943-483d-b678-b809bf464c30, and https://covid19.cog.sanger.ac.uk/submissions/release1/haniffa21.processed.h5ad.

## 1 Introduction

Single-cell RNA sequencing (scRNA-seq) has become a key method for elucidating complex biological processes through cell-level gene expression profiling. This high-resolution approach provides novel insights into various diseases, including COVID-19 (Stephenson *et al.* 2021), autism (Velmeshev *et al.* 2019), Alzheimer’s disease (Murdock and Tsai 2023), and cancers (Lei *et al.* 2021). It is instrumental in characterizing cellular heterogeneity, identifying rare cell populations, elucidating cellular interactions, and pinpointing molecular signatures associated with specific cellular lineages (González-Silva *et al.* 2020, Saviano *et al.* 2020, Longo *et al.* 2021). With the rapid decrease in the cost of scRNA-seq over the years, it has been applied to a growing number of individuals and studies, leading to a significant expansion in cohort size and data volume.

A fundamental research question in scRNA-seq revolves around understanding how gene expression in specific cell subpopulations is associated with variables of interest, such as disease status (Andrews *et al.* 2021). The statistical procedure used to identify these “signature” genes, or genes with differential expression (DE), is commonly referred to as DE analysis. These signature genes can offer insights into underlying biological processes and disease mechanisms and may serve as potential biomarkers for disease diagnosis, prognosis, and treatment selection.

Over the past few years, numerous DE analysis methods, such as SCDE (Kharchenko *et al.* 2014), MAST (Finak *et al.* 2015), scDD (Korthauer *et al.* 2016) and ZINB-WAVE (Risso *et al.* 2018), have been developed to compare different cell groups and identify cell subset- or condition-specific signatures [see the latest review by Guo *et al.* (2024)]. Most of these methods focus on identifying cell-level differential signatures by comparing expression patterns between groups of cells from one or a few biological replicates. However, with the decrease in sequencing costs, scRNA-seq studies now often include an increasing number of biological replicates (Velmeshev *et al.* 2019, Stephenson *et al.* 2021, Smajić *et al.* 2022, Mitchell *et al.* 2023), allowing for the identification of individual-level-differential signatures. Nevertheless, scRNA-seq studies involving multiple individuals introduce an additional layer of biological variability, ie, individual-to-individual variability, upon cell-to-cell variability within the same individual. This multi-layer variability complicates DE analysis, and most existing DE methods may not be sufficiently flexible to accommodate individual-to-individual variability. Pooling cells from different subjects and treating the cells as independent observations can lead to a high number of false positives (Squair *et al.* 2021). Accounting for within-subject correlation using mixed-effects modeling, as implemented in MAST (Finak *et al.* 2015) and muscat (Crowell *et al.* 2020), also shows a compromise of type I error, potentially due to data sparsity. Therefore, novel DE approaches are essential to address this unique challenge posed by multi-individual scRNA-seq studies.

Several recent methods have been proposed to improve individual-level DE analysis in scRNA-seq studies (Crowell *et al.* 2020, Thurman *et al.* 2021, Zhang *et al.* 2022, Zhang and Guo 2022). One common approach aggregates cell-level data into pseudo-bulk samples, as seen with aggregateBioVar (Thurman *et al.* 2021), or applies bulk RNA-seq DE methods like edgeR (Robinson *et al.* 2010), DESeq2 (Love *et al.* 2014), and limma-voom (Law *et al.* 2014) to these pseudo-bulk samples. However, pseudo-bulk methods discard important distributional features, limiting their ability to detect changes such as shifts in variance. Recent methods like IDEAS (Zhang *et al.* 2022) and BSDE (Zhang and Guo 2022) analyze the full distributional characteristics of gene expression using distance metrics, but they are computationally intensive and may not scale well to large datasets. IDEAS and BSDE typically take more than a day to analyze DE genes in a scRNA-seq study when using a personal computer without high-performance computing resources. Thus, there remains a need for new methods that combine effective false positive control, computational efficiency, and robust performance across diverse DE settings.

In this work, we introduce a novel method called DiSC for **d**ifferential expression analysis of **i**ndividual-level **sc**RNA-seq data. DiSC directly extracts distributional characteristics from gene expression data, tests these characteristics jointly using an omnibus-*F* statistic, and controls the false discovery rate (FDR) through a permutation-based procedure. Due to the simplicity of our procedure, DiSC is more than two orders of magnitude faster than IDEAS in real-world analyses, without compromising FDR control or statistical power.

## 2 Materials and methods

### 2.1 Method description

DiSC conducts DE analysis at the individual level for each cell subpopulation, following the assignment of cells to subpopulations using a cell clustering tool of choice (Kiselev *et al.* 2017, Butler *et al.* 2018). Although DiSC was primarily developed for scRNA-seq expression data, its application can extend to other single-cell expression datasets generated by technologies such as cytometry by time of flight (CyTOF). In the subsequent sections, we focus on the description of DiSC in the context of scRNA-seq expression data.

Suppose, for a given cell subpopulation, the scRNA-seq expression matrix $X=X_{M\times C}$ contains the transcript counts for M genes and a total of $C=\sum_{j=1}^{N}C_{j}$ cells from N individuals, with $C_{j}$ cells from the j-th individual (j=1, 2, ⋯, N). Let $x_{i,j,c}$ indicate the count for gene i (i=1, 2, ⋯, M) in the cell c ($c=1,2,\ldots ,C_{j}$) from the individual j (j=1, 2, ⋯, N). Furthermore, define $X_{i,j+}=[x_{i,j,1},x_{i,j,2},\ldots ,x_{i,j,C_{j}}]$, a row vector of a length $C_{j}$, to be the individual-level expression vector of gene i, comprising all cells from the individual j. Namely,

$$X_{M\times C}=[\begin{array}{ccccccccccccc} x_{1,1,1} & x_{1,1,2} & \cdots & x_{1,1,C_{1}} & x_{1,2,1} & x_{1,2,2} & \cdots & x_{1,2,C_{2}} & \cdots & x_{1,N,1} & x_{1,N,2} & \cdots & x_{1,N,C_{N}}\\ x_{2,1,1} & x_{2,1,2} & \cdots & x_{2,1,C_{1}} & x_{2,2,1} & x_{2,2,2} & \cdots & x_{2,2,C_{2}} & \cdots & x_{2,N,1} & x_{2,N,2} & \cdots & x_{2,N,C_{N}}\\ \vdots & \vdots & \ddots & \vdots & \vdots & \vdots & \ddots & \vdots & \ddots & \vdots & \vdots & \ddots & \vdots \\ x_{M,1,1} & x_{M,1,2} & \cdots & x_{M,1,C_{1}} & x_{M,2,1} & x_{M,2,2} & \cdots & x_{M,2,C_{2}} & \cdots & x_{M,N,1} & x_{M,N,2} & \cdots & x_{M,N,C_{N}} \end{array}]=[\begin{array}{cccc} X_{1,1+} & X_{1,2+} & \cdots & X_{1,N+}\\ X_{2,1+} & X_{2,2+} & \cdots & X_{2,N+}\\ \vdots & \vdots & \ddots & \vdots \\ X_{M,1+} & X_{M,2+} & \cdots & X_{M,N+} \end{array}]$$

Denote the $Y_{N\times p}$ and $Z_{N\times q}$ as the design matrix of p variable(s) of interest and the design matrix of q covariates required to be adjusted for, respectively. The aim of DiSC is to test the association between the individual-level variable(s) of interest, $Y_{N\times p}$, and the scRNA-seq expression vectors at the individual level, $X_{i,1+},X_{i,2+},\ldots ,X_{i,N+}$, for each gene i, while adjusting for the covariates $Z_{N\times q}$.

**Figure 1.**
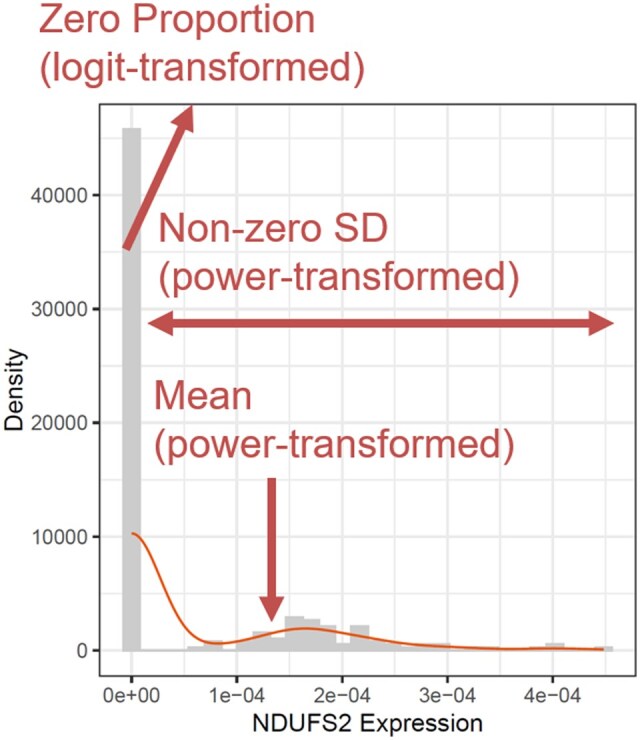
A schematic diagram illustrating the key steps of distributional feature extraction and transformation of NDUSF2 expression in CD8+ T cells from a COVID-19 patient using DiSC.

To achieve this goal, DiSC first normalizes the gene expression count matrix X column-wisely by dividing each cell’s transcript counts by the total transcript count within that cell. Let $X_{M\times C}^{'}$ be the normalized gene expression matrix. DiSC then extracts K features, denoted by $w_{i,j,k}$ (k=1, ⋯, K), from the normalized expression vector $X_{i,j+}^{'}$ for gene i and individual j. These features jointly characterize the distribution of the cell-level expression within an individual. Specifically, we have

$$w_{i,j,k}=f_{k}({X^{'}}_{i,j+}^{T}),(i=1,\ldots ,M;j=1,\ldots ,N;k=1,\ldots ,K),$$

and

$$W_{i,k}={\left[w_{i,1,k},w_{i,2,k},\ldots ,w_{i,N,k}\right]}^{T},$$

where $f_{k}$ represents the feature extraction function to create the kth feature (distributional characteristics) and $W_{i,k}$ is a vector comprising the kth feature from N individuals for gene i. As a default, DiSC uses the proportion of zeros, the mean, and the standard deviation of non-zero part of the expression counts to characterize the cell-level expression distribution. More distributional characteristics of interest can be readily incorporated, such as higher-order moments, given the flexibility of an “omnibus” testing framework in DiSC (stated below). An illustration of the distributional feature extraction and transformation in DiSC is shown in Fig. 1.

Subsequently, DiSC transforms the distributional characteristics using some transformation functions and fits linear models to the transformed characteristics.

$$g_{k}({\left(W_{i,k}\right)}_{N\times 1})\equiv {\left(U_{i,k}\right)}_{N\times 1}=Y_{N\times p}{\left(\alpha_{i,k}\right)}_{p\times 1}+Z_{N\times q}{\left(\beta_{i,k}\right)}_{q\times 1}+{\left(\varepsilon_{i,k}\right)}_{N\times 1},$$

where $g_{k}$ is the data transformation function for feature k, $U_{i,k}$ denotes the transformed feature for gene i and feature k, $\alpha_{i,k}$ and $\beta_{i,k}$ are regression coefficients, and $\varepsilon_{i,k}$ represents errors. Currently, we use a logit transformation capped at ±7 for the feature of proportion of zeros, ie $g_{1}(w)=\max(\min(\log\left(w/(1-w)\right),7),-7)$. This restriction reduces the influence of outlier values when transforming extremely high or low proportions of zeros. For the features of the mean and the standard deviation of non-zero counts, we use a power transformation $g_{2}(w)=g_{3}(w)=w^{\rho },\rho =0.5$. Our analysis showed that different exponents yielded similar performance, while ρ=0.5 provided slightly more power and remained robust to outliers (Yang and Chen 2023). Additionally, other user-specified feature transformation functions can be readily incorporated into the omnibus testing framework of DiSC as shown below. Based on the linear models, the strength of the association between the transformed feature $U_{i,k}$ and the variable of interest Y is quantified using a traditional F statistic:

$$F_{k,i}=\frac{\frac{U_{i,k}^{T}(H_{Y,Z}-H_{Z})U_{i,k}}{p}}{\frac{U_{i,k}^{T}(I-H_{Y,Z})U_{i,k}}{n-p-q}},$$

where $H_{Y,Z}$ and $H_{Z}$ are the hat or projection matrices onto the column space of (Y,Z) and Z respectively, and I is the identity matrix.

Finally, DiSC derives an omnibus F-statistic, which serves as a composite measure for the association evidence across various distributional characteristics. The omnibus F-statistic takes the maximum of F-statistics for different distributional characteristics considered:

$$F_{O,i}=\underset{k}{\max}(F_{k,i}).$$

The omnibus F-statistic may not follow an F-distribution, thus permutation is needed to control the type I error and FDR. The steps of permutations have been described in details in our previous work (Yang and Chen 2022) and can be summarized as follows. When there are no covariates, DiSC permutes the rows of the variable of interest $Y_{N\times q}$ to derive the omnibus F-statistic under permutation, $F_{O,i}^{b},b=1,2,\ldots ,B$, where B is the total number of permutations. When covariates $Z_{N\times q}$ are present in the study, DiSC derive $F_{O,i}^{b}$ based on the Smith’s procedure (Winkler *et al.* 2014). Based on $F_{O,i}^{b}$ and $F_{O,i}$, the *p*-value for gene i can be obtained as

$$p_{i}=\frac{\sum_{b}I(F_{O,i}^{b}\geq F_{O,i})}{B+1},$$

where I(.) is the indicator function, which takes on 1 if the inside relationship is satisfied and 0 elsewhere. To derive the q-value or FDR-adjusted p-value, assume $F_{O,(i)}$ is the i-th largest omnibus F-statistic across the M genes, and denote

${\tilde{q}}_{\left(i\right)}=\frac{\max(\sum_{l,b}I(F_{O,l}^{b}\geq F_{O,(i)}),\;0.5)}{i\cdot B},\;\mathrm{and}\;q_{\left(i\right)}=\underset{j\geq i}{\min}{\tilde{q}}_{\left(j\right)}.$

Here, the pseudo-count 0.5 is used if none of the $F_{O,l}^{b}\geq F_{O,(i)}$.

### 2.2 Simulation strategies

#### 2.2.1 Real-world data-based global null study

To unbiasedly assess the performance of DE methods, ideally, real-world datasets should be used. However, the absence of known ground truth (ie, truly differential genes) complicates further statistical power evaluation. Nonetheless, real datasets can still be used to assess type I error and/or FDR control under the global null hypothesis by eliminating true signals through label shuffling, ensuring that any detected positives are false positives. We conducted a global null study using scRNA-seq data from a COVID-19 study (Stephenson *et al.* 2021) and an autism study (Velmeshev *et al.* 2019), shuffling disease labels [COVID-19 severity or autism spectrum disorder (ASD) vs. control] 1000 times. While detailed dataset descriptions are available in the original publications, a brief summary is provided below.

The COVID-19 study (Stephenson *et al.* 2021) investigated peripheral blood mononuclear cell (PBMC) responses to SARS-CoV-2 infection through single-cell multi-omics analysis. We analyzed CD4+ T cells at the Newcastle site (49 individuals), with approximately 12 subjects per disease severity group: healthy, mild, moderate, and severe/critical. The disease severity was treated as an ordinal variable and encoded as integer scores (0–3) for association testing. The autism study (Velmeshev *et al.* 2019) analyzed postmortem prefrontal cortex (PFC) and anterior cingulate cortex (ACC) tissue from autism spectrum disorder (ASD) patients and healthy controls, comprising 14 cell types. Nuclei were isolated and sequenced using the 10x Genomics platform. For our analysis, we focused on layer 2/3 excitatory neurons in the PFC of 13 ASD patients and 10 controls.

Following quality control and preprocessing, genes with >80% sparsity (expressed in <20% of cells) were removed. The final expression matrices comprised approximately 2,200 genes and 32,000 cells in the COVID-19 dataset and 8,000 genes and 8,600 cells in the autism dataset.

#### 2.2.2 Parametric model-based simulation

Subsequently, we assessed type I error and FDR control, statistical power, and computational efficiency using a parametric model-based simulation. Simulated data were generated based on a hierarchical zero-inflated negative binomial (ZINB) model, whose parameters were estimated at different levels: cells, individuals, and the population, using the scRNA-seq data from layer 2/3 excitatory neurons in the PFC region of the autism dataset described above (Velmeshev *et al.* 2019). The parameter estimation and data generation processes were adapted from simulation protocols employed in previous studies (Lun and Marioni 2017, Zhang *et al.* 2022, Zhang and Guo 2022) and are summarized as follows.

The cell-level ZINB parameters, including mean ($\mu_{i,j,c}$), dispersion ($\varphi_{i,j,c}$), and the probabilities of zeros ($\pi_{i,j,c}$), were obtained using a deep count autoencoder (DCA) (Eraslan *et al.* 2019). We continue our convention of using i,j,c to represent gene, individual, and cell, respectively. A logarithmic transformation was applied to mean and dispersion parameters, and a logit transformation was applied to the probabilities of zeros to facilitate modeling. The top 8000 frequent genes were selected for our simulations, corresponding to sparsity filtering at a threshold of ∼80%. Individual-level parameters, including mean ($\mu_{i,j}$), dispersion ($\varphi_{i,j}$), and probabilities of zeros ($\pi_{i,j}$), were then extracted based on the median of cell parameters from individual j after adjusting for size factors and applying the data transformations. The standard deviation of the mean across cells for each individual ($\sigma_{i,j}$) was also estimated. Based on the individual-level parameters, we fitted a multivariate normal model with population-level mean parameters ($\mu_{i},\;\varphi_{i},\;\pi_{i},\;\sigma_{i}$) and the variance-covariance matrix $\Sigma_{i}$.

Concerning data generation processes, parameters were initially generated at an individual level. Individual-level parameters, denoted by $\mu_{i}^{j}$, $\varphi_{i}^{j}$, $\pi_{i}^{j}$, $\sigma_{i}^{j}$ ($i=1,2,\ldots ,8000;j=1,2,\ldots ,N_{\mathrm{sim}}$), were sampled from the multivariate normal model described above. Additionally, a continuous covariate, $z_{i}^{j}$, was incorporated into the generation of mean parameters ($\mu_{i}^{j}$), with regression coefficients estimated based on the RNA integrity number in the autism study. Subsequently, individual-level parameters of randomly selected differentially expressed genes were associated with a binary variable. The signal density of differentially expressed genes was set at 5% for each of the three types of differential signals, where the binary variable affects expression mean, variance, and both mean and variance. Half of the individuals were categorized as “cases”, with differentially expressed genes in cases being upregulated or downregulated by f folds with an equal probability. At the cell level, mean parameters for individual j and cell c ($\mu_{i}^{j,c}$, $c=1,2,\ldots ,N_{\mathrm{cell}}$) were sampled from a normal distribution $N(\mu_{i}^{j},{\left(\sigma_{i}^{j}\right)}^{2})$. The dispersion ($\varphi_{i}^{j,c}$) and probabilities of zeros ($\pi_{i}^{j,c}$) were inherited from parameters $\varphi_{i}^{j}$ and $\pi_{i}^{j}$ for the individual j. Finally, these parameters were inversely transformed into their original scale using an exponential or inverse-logit function. Count data for each of the $N_{\mathrm{sim}}$ individuals, including 8,000 genes and $N_{\mathrm{cell}}$ cells, were generated using a ZINB random number generator from the “emdbook” package (Bolker 2008) in the R platform using the mean, dispersion, and probability of zeros parameters.

We evaluated the type I error rate and FDR control performance based on 1000 simulation runs under a global null scenario, where f=1, $N_{\mathrm{cell}}=375$, and $N_{\mathrm{sim}}=24$. These parameter values aligned with the sample size and the average sequenced cell number observed in the autism study. We assessed FDR control and power using 50 simulation runs under three predefined scenarios: (i) varying fold changes for differentially expressed genes, with f ranging from 1.1 to 1.5; (ii) different cell numbers for each individual, with $N_{\mathrm{cell}}$ ranging from 250 to 500; (iii) different sample sizes, with $N_{\mathrm{sim}}$ ranging from 10 to 100.

### 2.3 Performance evaluation and competing methods

Performance was evaluated based on the type I error rate and FDR control, statistical power and computational efficiency. For the type I error rate, we assessed it by averaging the proportions of false positive findings in equally expressed genes based on unadjusted raw p-values over simulation runs. The nominal α level of 5% was used for the type I error rate assessment. To assess FDR control, we used observed or empirical FDR, which is the average false discovery proportion (FDP) over simulation runs, where FDP=FP/max(TP+FP, 1). We used a target FDR level of 10% for the FDR control assessment. Under the global null setting, the empirical FDR is equal to the percentage of simulation runs with any positive findings, which coincides with the observed family-wise error rate. For the assessment of statistical power, we used the average true positive rates (TPRs) over simulation runs, where TPR=TP/(TP+FN), representing the proportion of true positive findings among differentially expressed genes. Power assessment was only performed after FDR control (10% target level) since this reflects the practice used in real data analysis.

We benchmarked DiSC against IDEAS (Zhang *et al.* 2022) and the DESeq2-based pseudo-bulk method (Love *et al.* 2014), as the latter is widely used in real-world analyses. Additionally, DiSC was compared with BSDE (Zhang and Guo 2022) and iDESC (Liu *et al.* 2023) under a simplified setting without covariates, as these methods could not explicitly adjust for covariates. In IDEAS, a negative binomial model, Wasserstein distance, and permutational multivariate analysis of variance (PERMANOVA) were used to compute *P*-values, and the *q*-value (Storey 2003) approach was employed to control FDR. DESeq2, BSDE, and iDESC were applied using their default parameters or as recommended in their documentation.

## 3 Results

### 3.1 Real-world data-based global null study

A real-world data-based global null study was conducted to estimate and compare the actual type I error rate and FDR with the nominal levels. DiSC and IDEAS effectively controlled the type I error and FDR around or under the nominal levels of 5% and 10%, respectively (Table 1). However, DESeq2 showed severe FDR inflation, averaging 33.7% and 27.9%, together with slightly inflated type I error rates of 6.25% and 5.67% for the autism and COVID-PBMC datasets, respectively. Additionally, DiSC outperformed IDEAS and DESeq2 in controlling the average number of false positive findings (Table 1).

**Table 1.** The observed type I error rates and false discovery rates (FDRs) of various methods based on the real-world data-based and parametric model-based global null study.^a^

Dataset	Methods	Type I error rate (%)	False discovery rate (%)	Average number of false positive findings
COVID-PBMC	DiSC	5.09 (4.74, 5.44)	7.8 (6.3, 9.6)	7.1 (2.8, 11.4)
	IDEAS	5.25 (4.70, 5.81)	9.2 (7.6, 11.2)	61.2 (44.9, 77.5)
	DESeq2	5.67 (5.42, 5.92)	27.9 (25.2, 30.8)	8.0 (5.9, 10.2)
Autism	DiSC	5.16 (4.90, 5.42)	8.5 (6.9, 10.4)	10.4 (2.5, 18.4)
	IDEAS	5.18 (4.89, 5.47)	9.0 (7.4, 10.9)	42.9 (24.9, 60.9)
	DESeq2	6.25 (5.89, 6.61)	33.7 (30.8, 36.7)	54.4 (38.9, 69.9)
Parametric model-based simulation	DiSC	5.01 (5.02, 4.99)	9.1 (7.5, 11.0)	0.1 (0.1, 0.1)
	IDEAS	5.02 (5.04, 5.00)	7.9 (6.4, 9.7)	0.1 (0.1, 0.1)
	DESeq2	6.36 (6.38, 6.35)	91.1 (89.2, 92.7)	5.9 (6.2, 5.6)

a The nominal levels used for assessing the control of type I error rate and FDR were set at 0.05 and 0.10, respectively. Data are presented in the format “mean (95% CI)”.

### 3.2 Results of parametric model-based simulation studies

**Figure 2.**
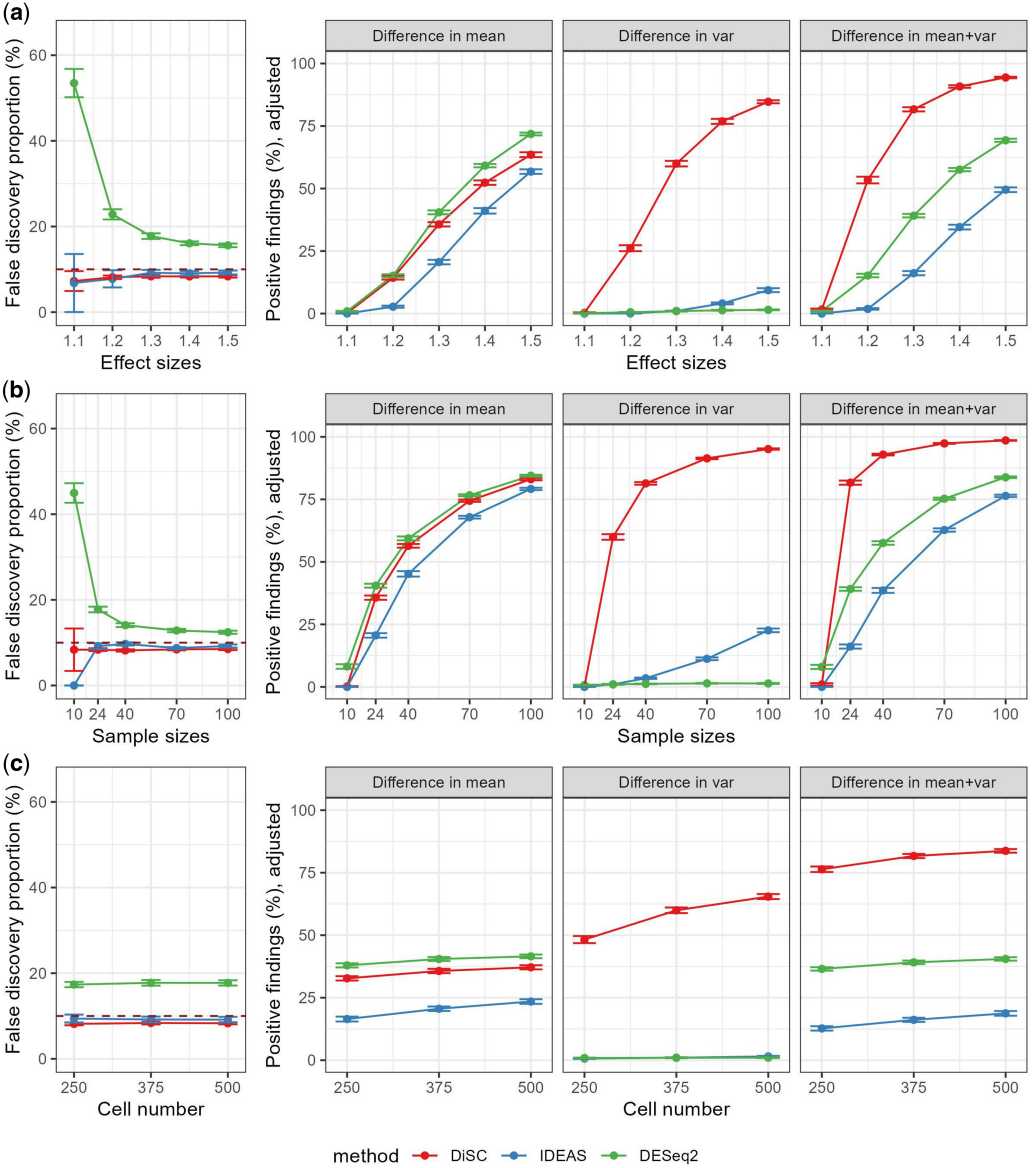
Performance of differential expression (DE) methods in parametric simulations with (a) increasing effect size (fold change), (b) sample size, and (c) the number of cells. After FDR control, genes with a *q*-value below 10% were considered positive. Error bars represent estimated standard errors. In each subfigure, the left panel presents the average false discovery proportions over simulations. The red dashed line indicates the 10% target level. The right panel presents the average true positive rates of different methods, by different types of DE.

Parametric model-based simulations were conducted to evaluate false positive control and compare statistical power among the competing methods under multiple settings, including various effect sizes, sample sizes, cell numbers, and types of differentially expressed genes. Under the global null hypothesis scenario, where we did not simulate differential signals, DiSC effectively controlled both type I error rates and FDRs at nominal levels of 5% and 10%, respectively (Table 1). The average false positives over simulation runs were considerably low at 0.1, significantly outperforming DESeq2 (Table 1). For scenarios with differentially expressed genes between cases and controls, DiSC demonstrated adequate false positive control across diverse settings and the average FDR ranged from 7.3% to 9.1% (Fig. 2). IDEAS also exhibited effective false positive control with an average FDR ranging from 0% to 9.7% across all settings (Table 1, Fig. 2). However, DESeq2 showed a poor performance in controlling false positives, with an average FDR ranging from 12.4% to 91.1% (Table 1, Fig. 2). This inflation was notably higher under the global null scenario or when the effect size and sample size were small.

For the statistical power comparison, DiSC demonstrated a high statistical power, or TPR, for different types of expression changes across settings, generally surpassing IDEAS (Fig. 2). Specifically, concerning genes with DE in the mean, DiSC showed a higher TPR compared to IDEAS across settings, although it was slightly lower than DESeq2. Since DESeq2 had a worse FDR control, the increased TPR may not be meaningful. In the case of genes with DE in variance, DiSC was consistently more powerful than competing methods across settings, including different effect sizes, sample sizes, and cell numbers per individual (Fig. 2). To achieve a comparable power, IDEAS required a higher sample size or effect size than DiSC. On the other hand, DESeq2 was not effective in detecting differences in variance as expected since aggregation will lose information on variance change. Notably, when both mean and variance changed, the statistical power of DiSC was further increased compared to scenarios where only mean or variance changed alone (Fig. 2). In contrast, when both mean and variance changed, the statistical power of DESeq2 and IDEAS remained the same or even relatively decreased compared to the case when the mean changed only.

Similar results were observed in the power comparison across DiSC, IDEAS, DESeq2, BSDE, and iDESC when no covariates were simulated (Table S2, available as supplementary data at *Bioinformatics* online). All methods effectively detected differential mean expression, but only DiSC and iDESC could efficiently identify DE in sample variance. DiSC achieved a higher TPR than IDEAS, BSDE, and iDESC for detecting mean differences and was the most powerful for detecting variance and joint mean-variance differences.

Additionally, a larger effect size and/or sample size increased the statistical power of DiSC to detect genes with differential mean and/or variance (Fig. 2). An increased number of cells per individual had a more significant effect on the power of DiSC for genes with differential variance, compared to its effect on genes with differential mean only (Fig. 2). A similar pattern was observed in DESeq2 and IDEAS, where their statistical power generally increased with larger sample sizes and effect sizes but showed less sensitivity to cell numbers.

### 3.3 Computational efficiency of DiSC on scRNA-seq data

**Figure 3.**
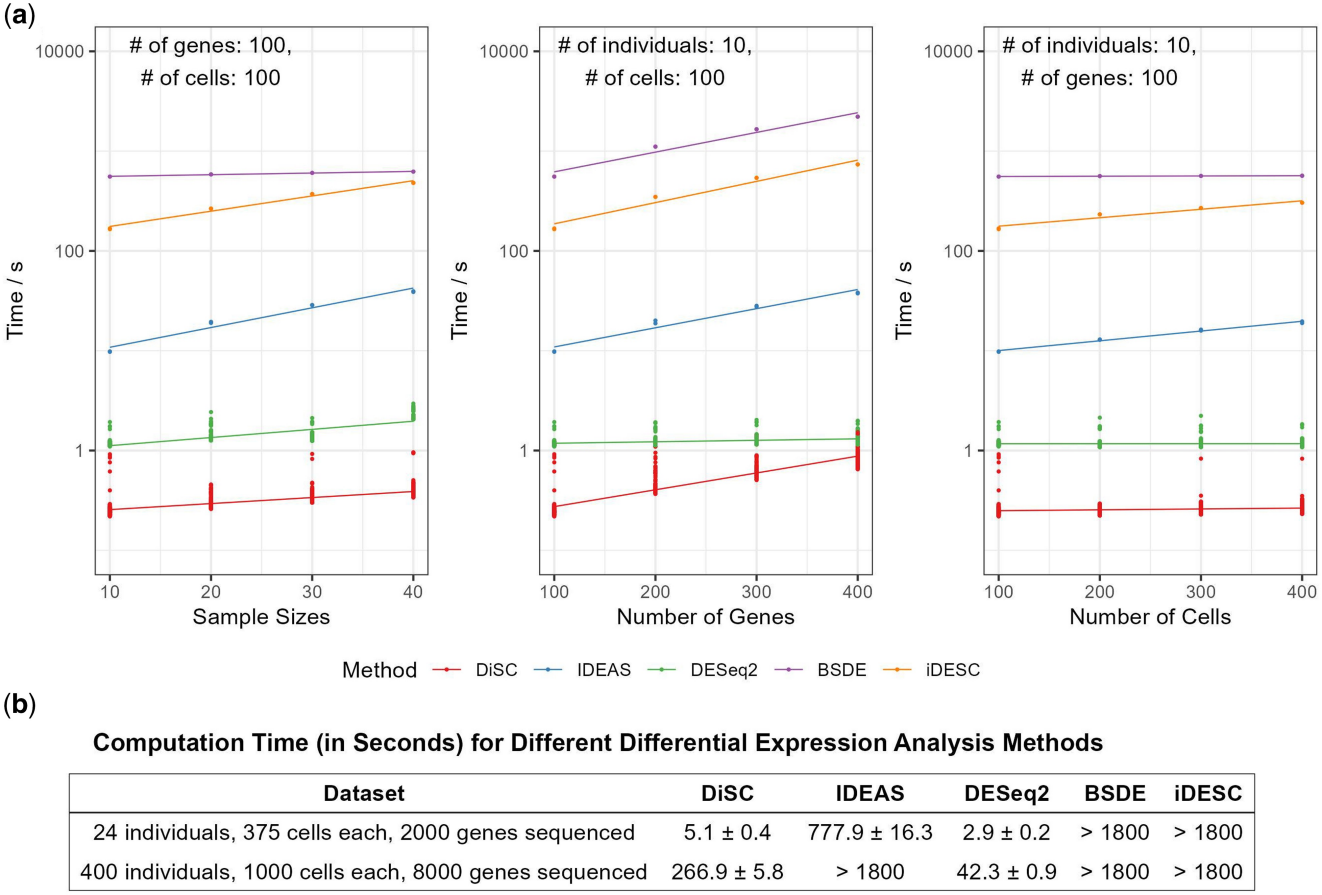
Computational efficiency of various methods. (a) Computational time (log scale) in relation to sample size, the total number of genes, and the number of cells per individual. (b) Computational time for scRNA-seq dataset with two typical sample sizes.

We evaluated the computational efficiency of different methods using a single thread on an AMD EPYC 7763 @ 2.4 GHz (Fig. 3). DiSC scaled efficiently with large sample sizes, maintaining a relatively stable computational time as the sample size (N) and cells per individual ($C_{j}$) increased (Fig. 3a). It analyzed DE genes in 5 seconds for a dataset with 2,000 genes, 24 individuals, and 9,000 cells, and in 4.5 minutes for 8,000 genes, 400 individuals, and 400,000 cells (Fig. 3b). DiSC was 100× faster than IDEAS, BSDE, and iDESC ($N_{\mathrm{perm}}=999$) for these practical sample sizes. Unlike IDEAS, BSDE, and iDESC, which typically require high-performance computing resources, DiSC runs efficiently on a laptop. Additionally, reducing permutations to 99 could further decrease DiSC’s runtime and memory usage without compromising power or FDR control (Table S1, available as supplementary data at *Bioinformatics* online).

### 3.4 DE analysis of genes potentially associated with COVID-19 severity in PBMCs

We applied DiSC to analyze 11 subtypes of PBMCs from the COVID-19 study (Stephenson *et al.* 2021) introduced above and conducted Gene Ontology (GO) enrichment analysis on the DE genes in CD4+ and CD8+ T cells. COVID-19 has posed a significant public health threat, with much of the research focused on understanding the immune system’s response, particularly the complex network of peripheral blood immune responses. In our analysis, we re-examined the immune responses in PBMCs using DiSC on a homogeneous cohort (n=49) from the Newcastle site within the COVID-19 study. The two PBMC subtypes, CD4+ and CD8+ T cells, were of particular interest as both have been well-recognized as key players in the immune response to SARS-CoV-2 infection (Tay *et al.* 2020).

**Figure 4.**
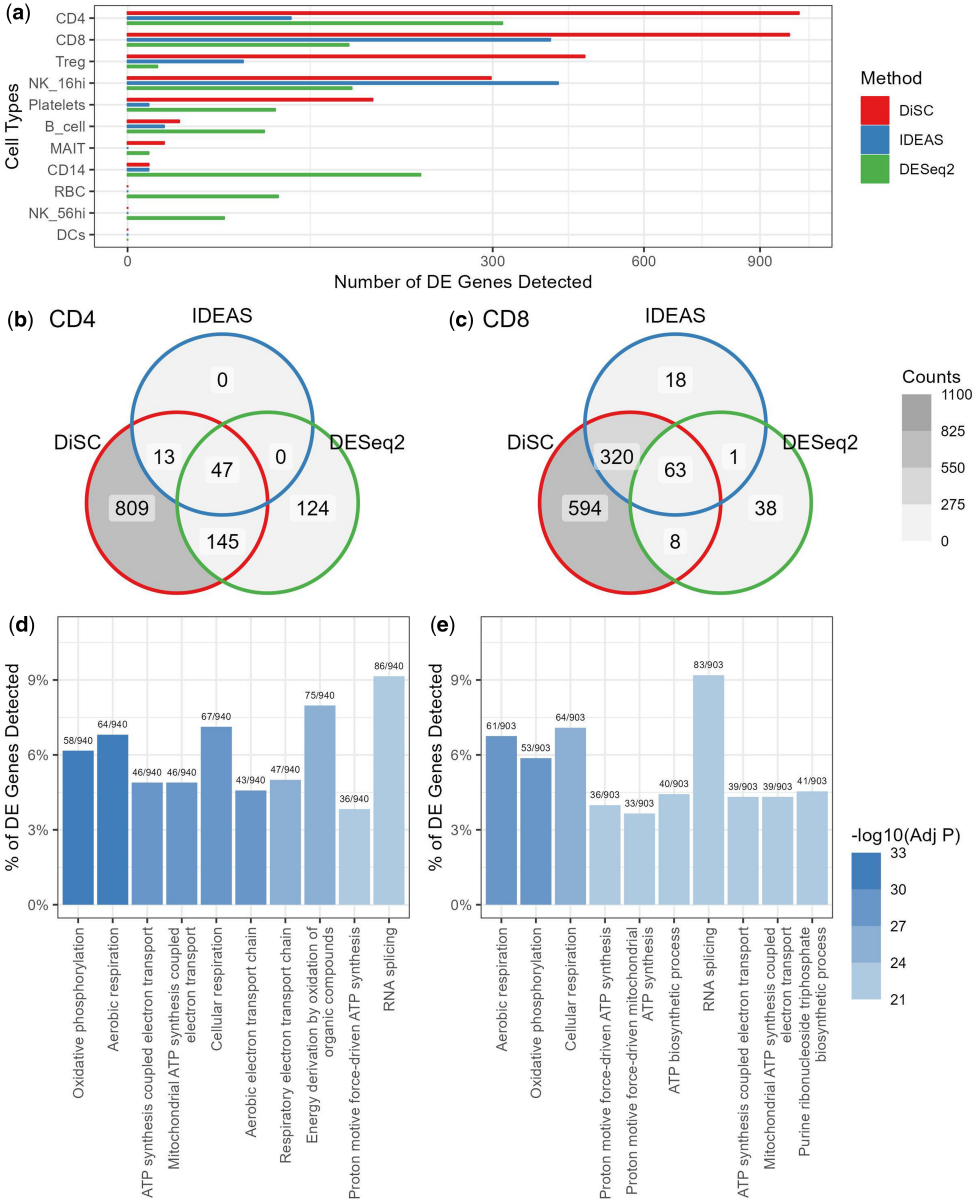
COVID-19-associated gene discovery. (a) The number of differentially expressed (DE) genes potentially associated with COVID-19 severity identified by different methods across 11 types of peripheral blood mononuclear cells. A 10% false discovery rate threshold was used. Abbreviations of cell types: CD4, CD4+ T cells; CD8, CD8+ T cells; Treg, regulatory T cells; NK_16hi, natural killer cells (CD16 high); B_cell, B cells; MAIT, mucosal-associated invariant T cells; CD14, CD14+ monocytes; RBC, red blood cells; NK_56hi, natural killer cells (CD56 high); DCs, dendritic cells. (b and c) The number of COVID-19-associated DE genes commonly discovered by each pair of methods in CD4+ (b) and CD8+ T cells (c). Overlap across methods suggests the potential robustness of the findings. (d and e) The top 10 significantly enriched Gene Ontology (GO) biological process terms in GO enrichment analysis for DE genes identified by DiSC in CD4+ (d) and CD8+ T cells (e).

After standard preprocessing including quality control and cell type annotation, we performed individual-level DE analyses using different competing methods, adjusting for covariates and multiple testing. At an 10% FDR threshold, DiSC identified more significant genes potentially associated with COVID-19 severity than IDEAS and DESeq2 in five out of the 11 cell types (Fig. 4a). IDEAS identified the most signature genes in one cell type. DESeq2 detected more genes in four cell types than both DiSC and IDEAS; however, this result should be interpreted with caution as its FDR may be inflated (Table 1).

Among the DE genes identified by DiSC in CD4+ and CD8+ T cells, a high proportion were also corroborated by IDEAS and DESeq2 (Fig. 4b and c). Specifically, DiSC re-identified 100% (60/60) of the DE genes detected by IDEAS and 61% (192/316) of those detected by DESeq2 in CD4+ T cells. In CD8+ T cells, DiSC re-identified 95% (383/402) of the IDEAS-detected DE genes and 65% (71/110) of those identified by DESeq2. Moreover, GO enrichment analysis revealed many significant biological processes associated with the DE genes identified by DiSC in CD4+ (Fig. 4d) and CD8+ T cells (Fig. 4e). The top 10 significantly enriched GO terms in both cell types were generally linked to energy metabolism. Similar results were observed when GO enrichment analysis was performed exclusively on the DE genes uniquely identified by DiSC. This suggests that SARS-CoV-2 infection may induce adaptations or dysfunction in the mitochondrial metabolic pathways of CD4+ and CD8+ T cells, a phenomenon that has been widely recognized in previous studies (Ajaz *et al.* 2021, Wik and Skålhegg 2022).

### 3.5 DE analysis of genes potentially associated with Alzheimer’s disease (AD)

Subsequently, we applied DiSC to analyze AD-associated DE genes in the Seattle Alzheimer’s Disease Brain Cell Atlas (SEA-AD) dataset (Gabitto *et al.* 2024). AD, a leading cause of dementia, is a progressive neurodegenerative disorder characterized by cognitive decline and memory loss. The SEA-AD project aims to elucidate the cellular, molecular, and epigenomic mechanisms underlying AD by integrating neuropathology, single-cell and spatial genomics, and longitudinal clinical metadata. This study utilized single-nucleus RNA sequencing to profile the middle temporal gyrus (MTG), a brain region involved in language processing, semantic memory, and visual perception. The dataset included approximately 1.4 million cells across 24 cell types from 88 donors, comprising 41 individuals with dementia and 47 cognitively normal controls.

**Figure 5.**
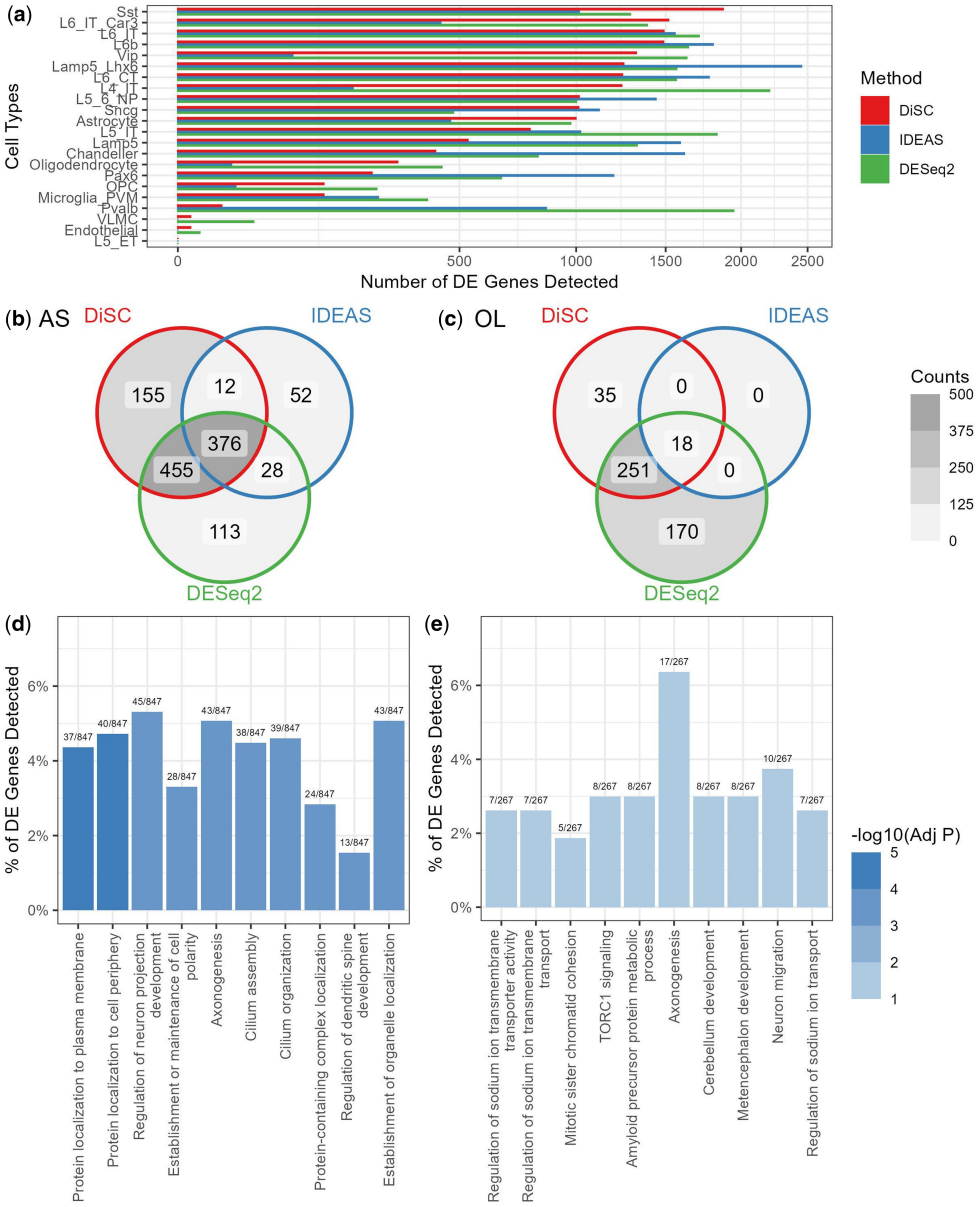
Identification of differentially expressed (DE) genes associated with Alzheimer’s disease (AD) using DiSC. (a) Number of DE genes potentially associated with AD identified by different methods across 22 neural cell subtypes, using a 10% false discovery rate threshold. (b and c) Overlap of AD-associated DE genes identified by each pair of methods in astrocytes (b) and oligodendrocytes (c). (d and e) The top 10 significantly enriched Gene Ontology (GO) biological process terms from GO enrichment analysis for DE genes identified by DiSC in astrocytes (d) and oligodendrocytes (e).

As in the COVID-19 PBMC dataset, we conducted similar analyses on the SEA-AD dataset, including preprocessing, DE analysis with the adjustment for confounders and multiple testing, and GO enrichment analysis in astrocytes and oligodendrocytes. DiSC identified more DE genes than IDEAS in 9 of 22 cell types and detected DE genes in VLMC and endothelial cells, where IDEAS did not identify any (Fig. 5a). DESeq2 identified more DE genes than both DiSC and IDEAS in 10 cell types and fewer genes in 11 cell types (Fig. 5a). However, our simulations showed that DESeq2 may not adequately control false positives in some settings, and therefore, the additional genes identified require further validation. A high proportion of DiSC-identified DE genes overlapped with those identified by IDEAS and DESeq2. Specifically, in astrocytes and oligodendrocytes, DiSC re-discovered 81.3% (843/1036) and 61.3% (269/439) of DE genes, respectively (Fig. 5b and c). These patterns were consistent with those observed in the COVID-19 dataset. To compare DiSC with BSDE and iDESC, we also performed DE analyses without covariate adjustment. DiSC identified more DE genes than BSDE and iDESC in 15 and 12 of the 22 cell types, respectively (Fig. S1, available as supplementary data at *Bioinformatics* online).

GO enrichment analysis of DiSC-discovered DE genes highlighted key biological processes potentially linked to AD in astrocytes and oligodendrocytes (Fig. 5d and e). These two cell subtypes have gained significant research interest for their roles in AD pathophysiology (Cai and Xiao 2016, Preman *et al.* 2021, Sadick *et al.* 2022, Kim *et al.* 2024, Kedia and Simons 2025), and their activation or loss in abundance has been reported to be associated with AD progression (Gabitto *et al.* 2024). In astrocytes, we found cilium assembly and organization might contribute to neuroinflammation and synaptic regulation, as primary cilia dysfunction has been associated with impaired signal transduction in AD (Preman *et al.* 2021). In oligodendrocytes, amyloid precursor protein metabolic processes and axonogenesis were enriched, both of which are closely linked to AD pathology (Cai and Xiao 2016). Additionally, the enrichment of sodium ion transport regulation aligns with evidence that oligodendrocyte dysfunction in AD disrupts ionic homeostasis and neuronal excitability (Kedia and Simons 2025).

### 3.6 Application of DiSC to DE analysis of CyTOF data in stage III melanoma

Although our method development is motivated by scRNA-Seq data, it can be applied to single-cell datasets generated from other single-cell technologies such as CyTOF (cytometry by time of flight). CyTOF is an application of mass cytometry to quantify the expression of pre-selected protein markers (up to ∼40) on the surface or interior of single cells (Spitzer and Nolan 2016). The CyTOF data is similar in format to the scRNA-seq data with a lower dimensionality and continuous measurements. One important application of CyTOF is to study the immunological response to a certain condition by profiling the expression of immune-related markers on the immune cell surfaces. Previously, we analyzed the CyTOF data to study the immunological responses to targeted therapy and immunotherapy in resectable Stage III melanoma (NeoACTIVATE trial) (Hieken *et al.* 2024). In the NeoACTIVATE trial, the patients were genotyped for BRAF mutation status (wild-type vs mutated). It is thus interesting to examine whether the BRAF mutation status is associated with the immune marker expression during the treatment course.

In the previous study (Hieken *et al.* 2024), we compared the marker expression between BRAF-wild-type and BRAF-mutated groups on the major T cell subsets (naïve, central memory, effector and EMRA CD4+ and CD8+ T cell, NKT cell, and gd T Cell) across four key time points during the treatment [baseline, after Cycle 1 neoadjuvant treatment (C1), after completion of neoadjuvant treatment (C3), and after operation (C4)]. To identify DE markers on different T cell subsets, we previously aggregated the marker expression within an immune cell subset using the population mean, followed by linear regression analyses. Although some meaningful trends were observed in this study, none of the markers survived FDR control at an 10% level due to a small sample size (n=30).

**Figure 6.**
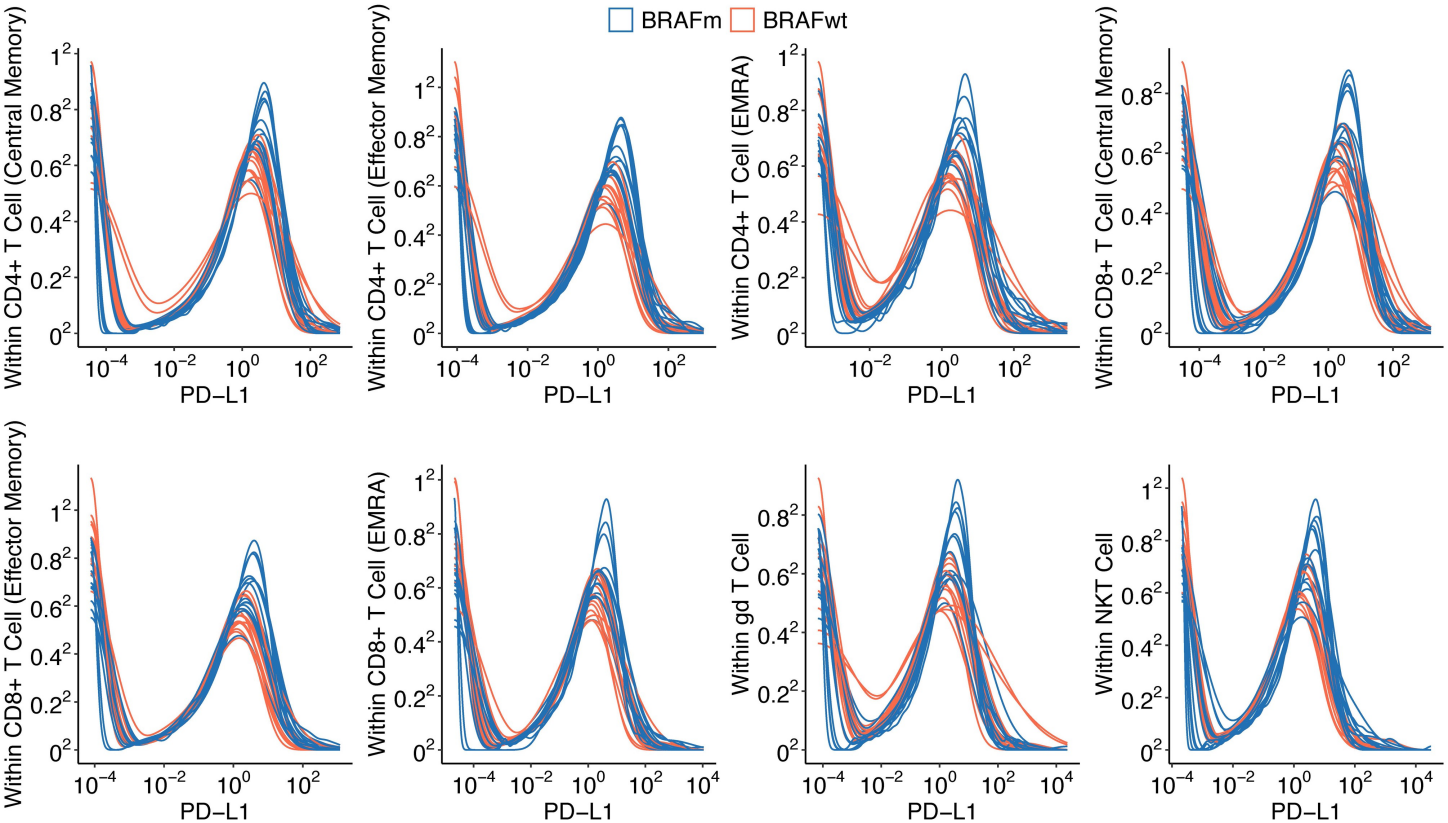
Distribution of cell-level PD-L1 expression within each individual at timepoint C1 (after Cycle 1 neoadjuvant treatment), stratified by T cell subpopulations and BRAF genotype. BRAF genotype status: BRAFm for BRAF-mutant and BRAFwt for BRAF wild-type.

In the present study, we re-analyzed the data using the DiSC approach and our novel approach helped recover statistically significant immune markers. While the aggregation-based method identified no significant markers at 10% FDR level in any of the T cell subsets and time points, DiSC identified many immune-related markers that were differentially expressed on various T cell subsets at different time points (Table S3, available as supplementary data at *Bioinformatics* online). Interestingly, we observed a widespread upregulation of programmed cell death ligand 1 (PD-L1) expression on T cell subsets (8 out of the 10 subsets) at the C1 time point (Fig. 6). PD-L1 is an immune checkpoint protein expressed on various cells, including cancer cells, that binds to PD-1 receptors on T cells to suppress their activity, allowing cancer cells to evade immune detection (Yamaguchi *et al.* 2022, Yi *et al.* 2022). PD-L1 expression also serves as a prognostic marker, often associated with more aggressive tumors, and anti-PD-1/PD-L1 therapies have significantly improved outcomes in cancers such as melanoma (Patel and Kurzrock 2015, Yamaguchi *et al.* 2022). Upregulation of PD-L1 expression has also been widely reported in association with BRAF mutations (Rosenbaum *et al.* 2016, Feng *et al.* 2019, Siraj *et al.* 2021).

## 4 Discussion

This study introduces DiSC, a novel method for conducting individual-level DE analysis on scRNA-seq datasets, tailored for multi-individual studies. Designed as a flexible and statistically robust protocol, DiSC extracts key distributional characteristics from cell-level expression data, jointly tests these features using an omnibus-F statistic, and maintains adequate control over type I error and FDR through a permutation-based procedure.

To retrieve robust DE signatures in scRNA-seq studies, an ideal individual-level DE analytical method should meet several key criteria. First, it should appropriately model variability by simultaneously accounting for gene expression variability across cells within the same individual (Gupta and Kuznicki 2020) and the biological variability across individuals within the study population. Since scRNA-seq captures a distribution of gene expression for each individual, the method should be capable of detecting shifts in the entire distribution, including changes in presence, mean, variance, or potentially higher-order moments of the expression distribution. Furthermore, it should control for false positives without sacrificing much statistical power. Finally, the method should also be computationally efficient to scale with the rapidly increasing sample sizes in scRNA-seq studies (Soneson and Robinson 2018, Andrews *et al.* 2021).

DiSC shows promising potential as a DE analysis method for multi-individual scRNA-seq studies. Our simulation studies demonstrate that DiSC effectively addresses biological variability at both cell and individual levels and appropriately controls false positives (Table 1, Fig. 2). Proper control of type I error and false positives is crucial for validating a DE method before its real-world application. Previous studies have highlighted the issue of type I error inflation of cell-level DE analyses, including methods such as scDD (Korthauer *et al.* 2016), ZINB-WAVE (Risso *et al.* 2018), and MAST (Finak *et al.* 2015), when not adequately addressing individual-level biological variability (Thurman *et al.* 2021, Zhang *et al.* 2022). One approach to adapt these methods for multi-individual scRNA-seq studies is to introduce a random effect to account for individual-level biological variability, as seen in muscat (Crowell *et al.* 2020) and the mixed-effect mode in MAST (Finak *et al.* 2015). The muscat framework provides several strategies to perform mixed-effect modeling using existing DE tools on either raw counts or variance-stabilized data. However, mild inflation of the type I error rate has still been observed, and such inflation persists even with increased sample sizes in real data (Zhang *et al.* 2022).

Alternatively, previous research has recommended applying bulk RNA-seq DE tools, such as DESeq2, to pseudo-bulk samples (Squair *et al.* 2021). The DESeq2-based pseudo-bulk approach has been shown to better account for individual-level biological variability, improve statistical power, and significantly reduce false discoveries under global null hypotheses (Squair *et al.* 2021). However, the FDR of pseudo-bulk analyses was not explicitly assessed or compared to nominal levels in that study (Squair *et al.* 2021). Our simulations indicate that while the average number of false discoveries remains low, DESeq2-based pseudo-bulk analysis may exhibit significant FDR inflation when signals are not strong (Li *et al.* 2022) (Table 1). Another study (Nguyen *et al.* 2023) demonstrated that pseudo-bulk methods can effectively detect DE genes and control false discoveries in specific scenarios. However, their evaluation approaches were distinct from the present work. Taken together, these findings underscore the need for further studies to validate the reliability of FDR control in pseudo-bulk methods under various conditions before their application to real-world DE analyses of scRNA-seq data.

Additionally, DiSC demonstrates high statistical power for detecting various types of DE (Fig. 2). scRNA-seq, as an advancement over bulk RNA sequencing, provides insights into cellular heterogeneity and gene expression distributions within cell subpopulations (Longo *et al.* 2021). In real-world data, compound distributional changes—such as simultaneous shifts in both mean and variance—could be as prevalent as the mean shifts alone. One major advantage of scRNA-seq is its ability to capture these complex distributional changes. Our simulations show that DiSC can more effectively leverage this advantage by detecting DE genes with both mean and variance changes, outperforming competing methods (Fig. 2).

Finally, DiSC exhibits high computational efficiency, making it scalable for large-scale study cohorts. DE analysis in multi-individual scRNA-seq studies can be computationally burdensome. More complex models, such as mixed-effect models, can offer higher flexibility and statistical power, but they are computationally demanding and usually prone to convergence issues. The trade-off between model complexity and computational efficiency has been an ongoing debate (Crowell *et al.* 2020). DiSC strikes a good balance, maintaining both computational efficiency (Fig. 3a) and statistical power (Fig. 2b) as sample sizes increase.

Count data normalization is critical in scRNA-seq DE analysis (Andrews *et al.* 2021), as various factors can influence sequencing depth, including biological variability in RNA content per cell and technical factors during sequencing. DiSC currently uses total sum scaling normalization by dividing transcript counts by the total transcript count per cell. Our simulations showed that this simple strategy performed similarly to other normalization methods such as the trimmed mean of M-values from edgeR and the relative log expression (RLE) from DESeq2 (Table S4, available as supplementary data at *Bioinformatics* online). However, our approach may be overly simplistic as it does not fully consider the data characteristics such as excess zeros in scRNA-seq data. Optimizing normalization strategies for scRNA-seq DE analysis remains an important direction for future research.

In conclusion, we introduce DiSC as a novel DE analysis tool for individual-level scRNA-seq data. Our simulation studies demonstrate that DiSC effectively controls the type I error rate and FDR through a permutation-based procedure, exhibits high statistical power in detecting various types of differential gene expression, and is computationally efficient and scalable to the rapidly increasing sample sizes in scRNA-seq studies.

## Supplementary Material

btaf327_Supplementary_Data

## Data Availability

The expression matrix and metadata for the immune response of peripheral blood mononuclear cells to COVID-19 (Stephenson *et al.* 2021) were retrieved from https://covid19.cog.sanger.ac.uk/submissions/release1/haniffa21.processed.h5ad. The single-cell RNA sequencing expression matrix and metadata utilized in our simulations and analyses, which included both healthy controls and autism spectrum disorder patients (Velmeshev *et al.* 2019), were obtained from https://cells.ucsc.edu/autism/rawMatrix.zip. The expression matrix and metadata for the Seattle Alzheimer’s Disease Brain Cell Atlas (SEA-AD) dataset (Gabitto *et al.* 2024) can be retrieved from https://cellxgene.cziscience.com/collections/1ca90a2d-2943-483d-b678-b809bf464c30.
