# Supplementary material for: DiSC: a statistical tool for fast differential expression analysis of individual-level single-cell RNA-seq data
Source: Bioinformatics. 2025 May 30;41(6):btaf327. doi: 10.1093/bioinformatics/btaf327 (PMC12203077; doi:10.1093/bioinformatics/btaf327)
Supplement: btaf327_Supplementary_Data [file btaf327_supplementary_data.docx]

**DiSC: a Statistical Tool for Fast Differential Expression Analysis of Individual-level Single-cell RNA-seq Data**

**Lujun Zhang^1^, Lu Yang^2^, Yingxue Ren^3^, Shuwen Zhang^2^, Weihua Guan^1^, Chen Jun^2, †^**

1 Division of Biostatistics and Health Data Science, University of Minnesota, Minneapolis, MN, USA

2 Department of Quantitative Health Sciences, Mayo Clinic, Rochester, MN, USA

3 Department of Quantitative Health Sciences, Mayo Clinic, Jacksonville, FL, USA

† To whom (Chen.Jun2@mayo.edu) correspondence should be addressed.

**Supplementary materials**

Table S1. The robustness of DiSC $p$-values across permutation numbers. The table displays $p$-values computed by DiSC using different permutation numbers (49, 99, 499, and 999), adjusted for multiple testing correction based on the false discovery rate. It presents adjusted $p$-values for one randomly selected gene from five categories: adjusted $p$-values less than or equal to 0.001, 0.001 – 0.01, 0.01 – 0.05, 0.05 – 0.10, and greater than 0.10.


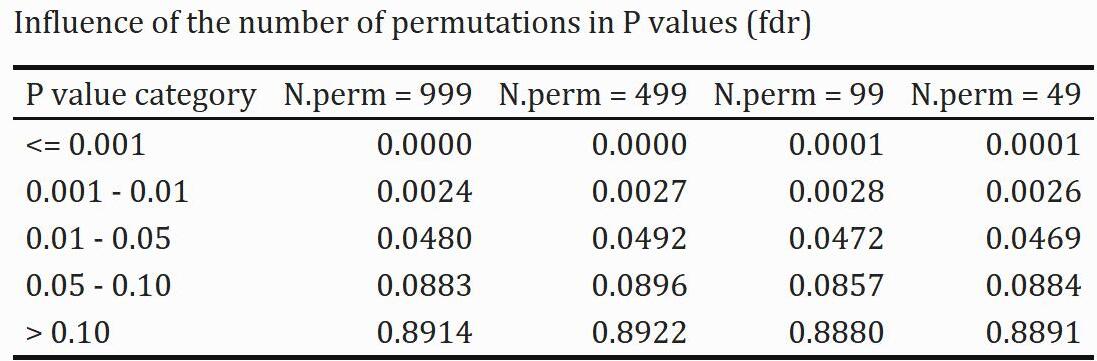


Table S2. Performance of five competing differential expression methods in parametric simulations. The simulations used an effect size of 1.3, 24 individuals, and 375 cells per individual, without covariate adjustment. Genes with q-values below 10% after FDR control were considered significant. Data are presented in the format “mean (95% CI)”. Abbreviations: DE, differential expression.

| Methods | Type I Error Rate (%) | False Discovery Proportions (%) | Positive Findings (%), DE in Mean | Positive Findings (%), DE in Variance | Positive Findings (%), DE in Both Mean and Variance |
| --- | --- | --- | --- | --- | --- |
| DiSC | 5.1  (4.8, 5.3) | 8.2  (7.6, 8.9) | 36.3  (35.2, 37.4) | 61.3  (60, 62.5) | 90.5  (90, 90.9) |
| IDEAS | 5.0  (4.8, 5.2) | 10.4  (9.1, 11.7) | 22.2  (20.6, 23.7) | 1.1  (0.9, 1.3) | 46.4  (45.2, 47.7) |
| DESeq2 | 6.4  (6.1, 6.7) | 17.6  (15.9, 19.3) | 40.3  (39.2, 41.4) | 0.9  (0.7, 1.1) | 61.4  (60.5, 62.3) |
| BSDE | 4.9  (4.6, 5.2) | 9.4  (8.5, 10.4) | 32.2  (31.3, 33.1) | 1.0  (0.8, 1.2) | 57.1  (56.3, 57.9) |
| iDESC | 8.9  (4.7, 13.1) | 25.7  (14.2, 37.2) | 27.2  (21.9, 32.5) | 31.0  (25.6, 36.4) | 75.3  (71.8, 78.8) |

Figure S1. Identification of differentially expressed (DE) genes associated with Alzheimer’s disease (AD) using DiSC. DE analyses were conducted without the adjustment for covariates. (a) Number of DE genes potentially associated with AD identified by different methods across 22 neural cell subtypes, using a 10% false discovery rate threshold. (b & c) Overlap of AD-associated DE genes identified by each pair of methods in astrocytes (b) and oligodendrocytes (c).


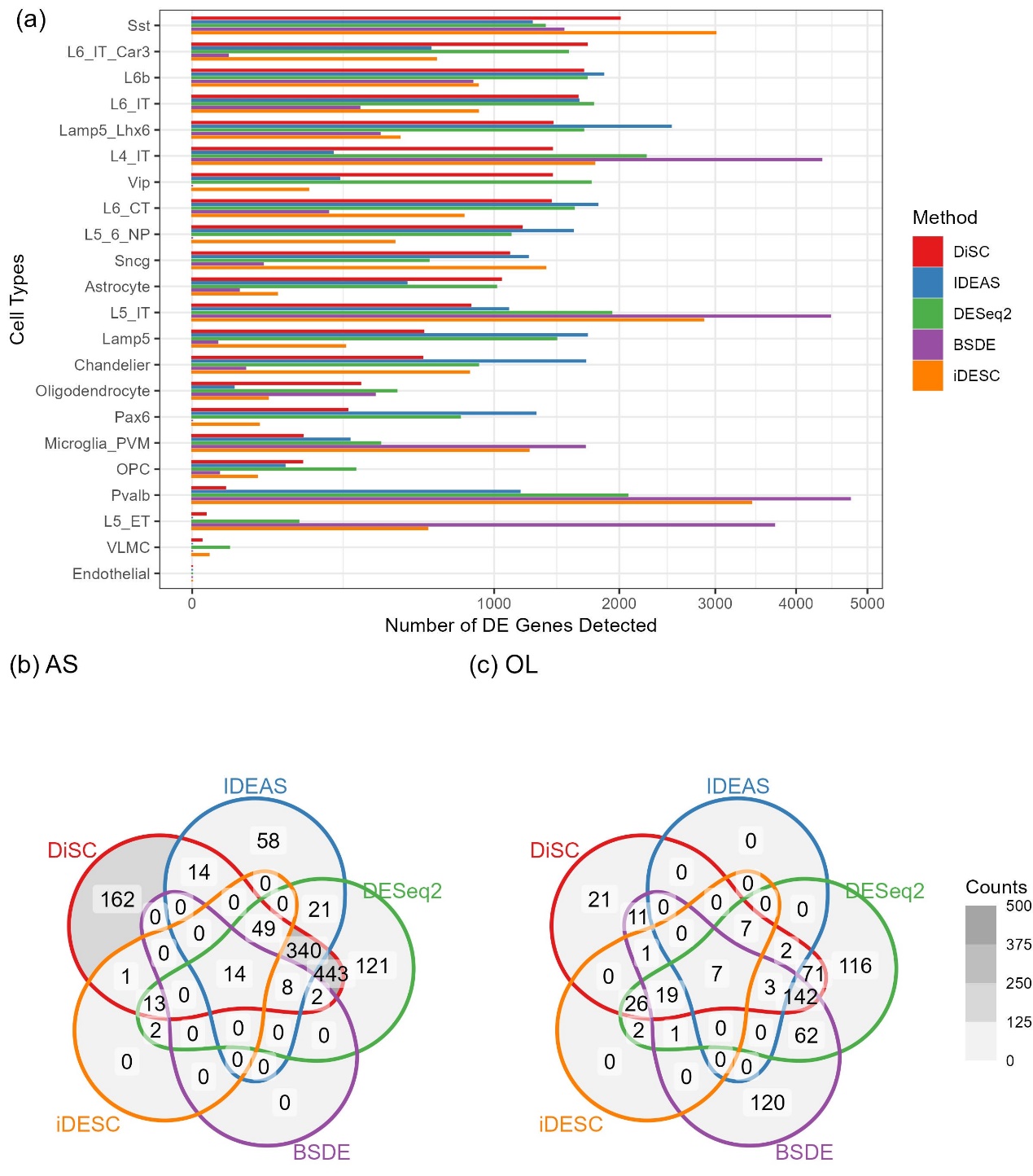


Table S3. Differential markers between wild-type and BRAF-mutated cells identified by DiSC within each cell type from the NeoACTIVATE trial [1] ($n=30$), with a 10% FDR. Different timepoints included: Baseline, at baseline; C1, after Cycle 1 neoadjuvant treatment; C3, after completion of neoadjuvant treatment; C4, after operation.

| Cell Types | Timepoints | | | |
| --- | --- | --- | --- | --- |
|  | Baseline | C1 | C3 | C4 |
| CD4+ T Cell (Central Memory) |  | CD11c, PD-L1 | CD183/CXCR3 |  |
| CD4+ T Cell (Effector Memory) |  | CD11c, PD-L1 |  |  |
| CD4+ T Cell (EMRA) |  | IgD, CD11c, CD27, HLA-DR, CD123/IL-3R, CD197/CCR7, CD66b, PD-L1, CD11a, PD-1 |  | CD19, CD123/IL-3R, PD-L1 |
| CD4+ T Cell (Naive) |  | CD11c, HLA-DR, CD123/IL-3R, CD11a, TIGIT |  | CD123/IL-3R, PD-1 |
| CD8+ T Cell (Central Memory) |  | CD11c, HLA-DR, CD123/IL-3R, PD-L1, PD-1, TIGIT |  | CD123/IL-3R, CD14, PD-1 |
| CD8+ T Cell (Effector Memory) |  | CD11c, PD-L1 |  | CD14 |
| CD8+ T Cell (EMRA) | CD27, CD194/CCR4, CD14, PD-1 | PD-L1 |  | CD123/IL-3R, CD14, PD-1 |
| CD8+ T Cell (Naive) |  | CD16, CD19, IgD, CD11c, CD27, HLA-DR, CD123/IL-3R, CD45RO, CD56/NCAM, CD66b, CD161, CTLA-4, CX3CR1, CD11a, PD-1, TIGIT |  | CD123/IL-3R, CD185/CXCR5, CD14, PD-1 |
| NKT Cell | PD-1 | PD-L1 |  | CD16, CD194/CCR4, CD14, PD-1 |
| gd T Cell |  | CD11c, CD45RA, HLA-DR, CD38, CD123/IL-3R, CD66b, CD161, PD-L1, PD-1, TIGIT | CD161 | CD123/IL-3R, CD14, CD161, PD-1, TIGIT |

Table S4. Influence of different normalization methods on true positive rate (%). We evaluated various normalization methods, including log2(x+1) transformation (no normalization), total sum scaling (TSS), the trimmed mean of M-values (TMM) and the relative log expression (RLE). Each method was applied with or without an adjustment for log median read depth at the individual level.

| Normalization Methods | Ture Positive Rate (%) by Differential Expression Type | | |
| --- | --- | --- | --- |
|  | mean DE | var DE | mean + var DE |
| **TSS, adjusted (implemented)** | **39.50** | **66.25** | **84.25** |
| no normalization | 40.75 | 70.25 | 86.75 |
| TSS | 35.50 | 53.50 | 80.75 |
| TMM | 33.00 | 41.75 | 75.00 |
| RLE | 39.75 | 67.00 | 84.50 |
| no normalization, adjusted | 40.25 | 69.50 | 86.50 |
| TMM, adjusted | 38.50 | 66.25 | 84.50 |
| RLE, adjusted | 38.75 | 65.75 | 84.25 |

**References:**

[1]. Hieken, T.J., et al., Neoadjuvant cobimetinib and atezolizumab with or without vemurafenib for high-risk operable Stage III melanoma: the Phase II NeoACTIVATE trial. Nature communications, 2024. 15(1): p. 1430-1430.
